# A dataset for medical instructional video classification and question answering

**DOI:** 10.1038/s41597-023-02036-y

**Published:** 2023-03-22

**Authors:** Deepak Gupta, Kush Attal, Dina Demner-Fushman

**Affiliations:** Lister Hill National Center for Biomedical Communications, National Library of Medicine, National Institutes of Health, Bethesda, MD USA

**Keywords:** Information technology, Scientific data

## Abstract

This paper introduces a new challenge and datasets to foster research toward designing systems that can understand medical videos and provide visual answers to natural language questions. We believe medical videos may provide the best possible answers to many first aid, medical emergency, and medical education questions. Toward this, we created the MedVidCL and MedVidQA datasets and introduce the tasks of Medical Video Classification (MVC) and Medical Visual Answer Localization (MVAL), two tasks that focus on cross-modal (medical language and medical video) understanding. The proposed tasks and datasets have the potential to support the development of sophisticated downstream applications that can benefit the public and medical practitioners. Our datasets consist of 6,117 fine-grained annotated videos for the MVC task and 3,010 questions and answers timestamps from 899 videos for the MVAL task. These datasets have been verified and corrected by medical informatics experts. We have also benchmarked each task with the created MedVidCL and MedVidQA datasets and propose the multimodal learning methods that set competitive baselines for future research.

## Background & Summary

One of the key goals of artificial intelligence (AI) is developing a multimodal system that facilitates communication with the visual world (i.e., images and videos) using a natural language query. In recent years, significant progress has been achieved due to the introduction of large-scale language-vision datasets and the development of efficient deep neural techniques that bridge the gap between language and visual understanding. Improvements have been made in numerous vision-and-language tasks, such as visual captioning^1,2^, visual question answering^3^, and natural language video localization^4–6^. In recent years there has been an increasing interest in video question-answering^7,8^ tasks, where given a video, the systems are expected to retrieve the answer to a natural language question about the content in the video. We argue that only predicting the natural language answer does not seem to reflect the real world, where people interact through natural language questions and expect to localize the moments from the videos to answer their questions. The majority of the existing work on video question answering (VQA) focuses on **(a)** open-domain applications by building the VQA datasets^8–10^ consisting of movies, TV shows, and games, and **(b)** retrieval^7–9^ of the natural language answers. With increasing interest in AI to support clinical decision-making and improve patient engagement^11^, there is a need to explore such challenges and develop efficient algorithms for medical language-video understanding.

**Fig. 1:**
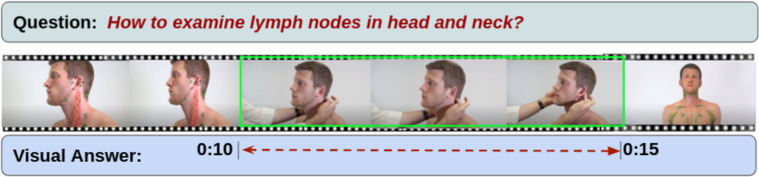
A sample example of a health-related question and its visual answer (temporal segment) from the video.

The recent surge in the availability of online videos has changed the way of acquiring information and knowledge. Many people prefer instructional videos to teach or learn how to accomplish a particular task with a series of step-by-step procedures. Medical instructional videos are more suitable and beneficial for delivering key information through visual and verbal communication at the same time in an effective and efficient manner. Consider the following medical question: “*how to place a tourniquet in case of fingertip avulsions?*” The textual answer to this question will be hard to understand and act upon without visual aid. To provide visual aid, we first need to identify the relevant video that is medical and instructional in nature. Once we find a relevant video, it is often the case that the entire video can not be considered as the answer to the given question. Instead, we want to refer to a particular temporal segment, or a sequence of moments, from the video, where the answer is being shown, or the explanation is being illustrated. The straightforward moment retrieval *via* an action, object, or attribute keyword may not uniquely identify the relevant temporal segment, which consists of the visual answer to the question. A more natural way to refer to the appropriate temporal segment (*c.f*. Figure 1) is via natural language question and video segment description, which requires a fine-grained semantic understanding of the video segment, segment description, and question.

Furthermore, the necessity of medical instructional videos is not only limited to the group having medical knowledge. But, it is also prevalent and necessary for the general public with limited or no medical knowledge. For example, queries such as “*how to check for skin/breast cancer?*” or “*how to reduce shoulder pain?*” are best addressed through multi-media education, such as videos, with clear visuals and instructions. In addition,^12^ notes that multi-media education is integral not only to delivering digital answers to consumer health questions but also empowers users to actively care for themselves. Also, in the context of the recent COVID-19 pandemic having access to credible health information in an understandable, digestible manner, like a medical instructional video, is necessary for informed health literacy.

**Fig. 2:**
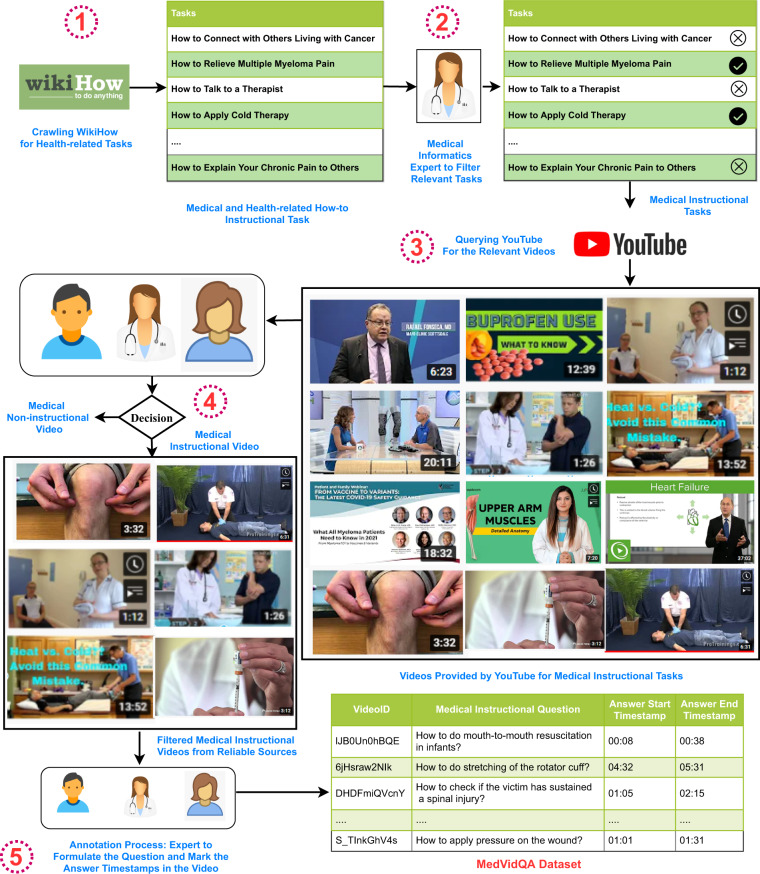
The schematic workflow of the MedVidQA dataset creation. Each step  is discussed in MedVidQA Data Creation.

**Fig. 3:**
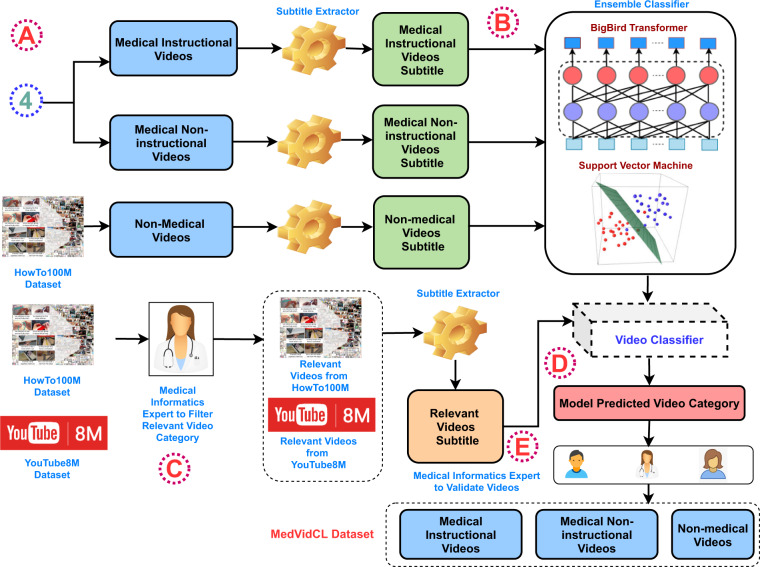
The schematic workflow of the MedVidCL dataset creation. It starts with the collection of medical instructional and non-instructional videos from step  of the MedVidQA dataset creation. Each step  is discussed in MedVidCL Data Creation.

Toward this, this work introduces the **Med**ical **Vid**eo **CL**assification (MedVidCL) and **Med**ical **Vid**eo **Q**uestion **A**nswering (MedVidQA) datasets^13^ for medical instructional video classification and question answering. The MedVidCL dataset contains a collection of 6,617 videos annotated into *‘medical instructional’*, *‘medical non-instructional’* and *‘non-medical’* classes. We adopted a two-step approach to construct the MedVidCL dataset. In the first step, we utilize the videos annotated by health informatics experts to train a machine learning model that predicts the given video to one of the three aforementioned classes. In the second step, we only use high-confidence videos and manually assess the model’s predicted video category, updating the category wherever needed. The MedVidQA dataset contains the collection of 3,010 manually created health-related questions and timestamps as visual answers to those questions from trusted video sources, such as accredited medical schools with an established reputation, health institutes, health education, and medical practitioners. We have provided a schematic overview of building the MedVidQA and MedVidCL datasets^13^ in Figs. 2, 3, respectively. We benchmarked the datasets by experimenting with multiple algorithms for video classification and video localization.

## Methods

### MedVidQA data creation

To create the MedVidQA dataset, we follow a systematic approach that involves the contributions of medical informatics experts at multiple stages. The detailed steps to build the dataset are as follows:

1. **Extraction of Medical and Health-related Tasks from wikiHow:** With an aim to obtain medical instructional videos that describe how to perform certain health-related activities, we first start by compiling an extensive list of health-related activities using wikiHow (https://www.wikihow.com/Main-Page) – an online resource that contains 235,877 articles on how to do a certain task for a variety of domains ranging from computer and electronics to philosophy and religion, structured in a hierarchy. We start with extracting the medical and health-related tasks from the wikiHow. We collected a total of 6,761 how-to tasks from the wikiHow ‘Health’ category.
2. **Identification of Relevant Health-related Tasks:** In the second step of the dataset creation, we filter the compiled collection extracted from wikiHow. A medical informatics expert reviews each wikiHow task and marks them as relevant or non-relevant from a medical instructional perspective. We keep only those tasks for which the textual answer will be hard to understand and act upon without visual aid such as “*how to relieve multiple myeloma pain*” or “*how to apply cold therapy*”. This procedure yields 1,677 medical and health-related instructional tasks. We have provided the distribution of the selected instructional tasks category in Fig. 7.
3. **Searching YouTube for The Relevant Videos:** To acquire the relevant videos, we use the task name as a query to search YouTube via its Data API (https://developers.google.com/youtube/v3). In order to collect only the most relevant videos, we only keep the top 4 videos returned by YouTube. We deduplicate videos based on YouTube IDs because some videos may appear in multiple health-related instructional tasks. However, if a video was uploaded multiple times or edited and re-uploaded, the dataset may still contain duplicates.
4. **Expert Annotation for Medical Instructional Videos:** Medical informatics experts further categorize the relevant medical instructional videos retrieved from YouTube searches. We perform this important step in our dataset creation because **(1)** videos retrieved by the YouTube search may not be instructional for particular medial queries, and **(2)** ensures the reliability of the medical videos. To identify the medical instructional videos in a pool of YouTube videos, we define medical instructional videos as follows: a medical instructional video should clearly demonstrate a medical procedure providing enough details to reproduce the procedure and achieve the desired results. The accompanying narrative should be to the point and should clearly describe the steps in the visual content. If a valid medical query is aligned with an instructional medical video, it should explain/answer the medical query with a demonstration, be a tutorial/educational video where someone (e.g., a *doctor* or a *medical professional*) demonstrates a procedure related to the medical query, or be a how-to video about the *medical query*. Medical instructional videos may target different levels of expertise, ranging from good Samaritans providing first aid to medical students learning a procedure or experienced clinicians interested in continuing medical education. For this study, we focus on the instructional medical videos that do not require medical education, i.e., the instructions should be at a level that is understandable and can be performed by a layperson. For example, if a nurse shows how to bandage a wound in an emergency, the video is instructional for a layperson. Conversely, if a doctor explains how to perform a specific surgical procedure, the video is instructional for professionals but not for the general public.
5. **Formulating Instructional Question and Visual Answer from Videos:** With the aim of formulating medical and health-related questions and localizing their visual answer in the videos, we start with the medical instructional videos annotated in the previous step of the dataset creation. A question is called instructional if the answer requires a step-by-step demonstration and description of the actions to be taken to achieve the goals. For many medical questions, the answer to the question is better shown than described in words, and the answer will be hard to understand and act upon without visual aid, e.g., *“how to perform a physical examination for breast abnormalities?”* Three medical informatics experts were asked to formulate the medical and health-related instructional questions by watching the given video and localizing the visual answer to those instructional questions by providing their timestamps in the video. We asked the annotators to create questions for which **(1)** answers are shown, or the explanation is illustrated in the video, **(2)** the given video is necessary to answer the question, and **(3)** the answer cannot be given as text or spoken information without visual aid.

### MedVidCL data creation

A video question-answering system that can provide visual answers to medical or health-related instructional questions must have the capability to distinguish between medical instructional and non-instructional videos related to the user’s questions. Towards building systems to perform this task efficiently and effectively, we created the MedVidCL dataset, which can be used to train a system that can distinguish among the medical instructional, medical non-instructional, and non-medical videos. The details of the approach to build MedVidCL dataset are described as follows:

(A) **Collecting Medical and Non-medical Videos**: With an aim to reduce the annotation efforts, we follow a two-step process to build the MedVidCL dataset. In the first step, we seek a high-confidence opinion on the video category from a pre-trained video classifier. In the second step, medical informatics experts validate the video category predicted by the video classifier. In order to train the video classifier, we begin with collecting medical and non-medical videos. We utilized the human-annotated 1,016 medical instructional and 2,685 medical non-instructional videos from MedVidQA dataset. To collect non-medical videos, we sampled 1,157 videos of non-medical categories (Food and Entertaining, Cars & Other Vehicles, Hobbies and Crafts, Home and Garden Tools, etc.) from the HowTo100M^14^ dataset, which is a large-scale YouTube video dataset with an emphasis on instructional videos. We perform a stratified split on this collection and used 80% videos for training, 10% for validation, and 10% for testing.
(B) **Building Video Classifier:** We focus on only coarse-grained (*medical instructional*, *medical non-instructional* and *non-medical*) categorization of the videos as opposed to the fine-grained (*walking*, *running*, *playing*, *standing*, etc.) video classification^15^ where the micro-level human activity recognition is the key to correctly categorizing the video. Therefore, we hypothesized it is possible to predict the coarse-grained category from the natural language subtitles of the video. Towards this, we propose an ensemble classifier that aggregates the predictions of deep learning and statistical classifiers. We used the support vector machine (SVM)^16^ with TF-IDF features as the statistical classifier in our ensemble learning setup, and we chose the pre-trained BigBird^17^ model as our deep learning classifier as BigBird is capable of accommodating longer sequences that are ubiquitous in video subtitles. We utilized the Hugging Face’s implementation (https://huggingface.co/google/bigbird-roberta-large) of the BigBird model. After extracting the English video subtitles using the Pytube library (https://pypi.org/project/pytube/), we fine-tuned four different pre-trained BigBird models, each with 1024 as the maximum token length on our training dataset. We also used early stopping to prevent overfitting the model. Since our training dataset has a skewed distribution of the classes, we consider penalizing the model in the training phase for the misclassification made for the minority class by setting a higher class weight and, at the same time, reducing the weight for the majority class. For the class *c* ∈ *C*, we set the weight ${w}_{c}=\frac{N}{| C| \times {N}_{c}}$, where *C* is the set of all classes in the dataset and *N* is the total number of samples in the dataset. *N*_*c*_ is the number of samples associated with class *c* in the dataset. We follow the Population Based Training (PBT)^18^ mechanism to jointly optimize a population of models and their hyperparameters to maximize performance. PBT inherits the idea of *exploitation* and *exploration* from genetic algorithms. In PBT, each member of the population *exploits* - taking into account the performance of the whole population, a member can decide whether it should abandon the current solution and focus on a more promising one - and *explores* - considering the current solution and hyperparameters, it proposes new ones to better explore the solution space. Following this, we fine-tune the BigBird with the PBT strategy (population size = 5) and consider the top-2 performing members of the population as final models. We used two different PBT strategies to train the BigBird model. In one strategy, we penalize the model and call their top-2 members of the population as ${{\rm{BigBird}}}_{{\rm{w/}}\;{\rm{weight}}}^{1}$ and ${{\rm{BigBird}}}_{{\rm{w/}}\;{\rm{weight}}}^{2}$. In another strategy, we train the BigBird models without penalizing them and call them ${{\rm{BigBird}}}_{{\rm{w/o}}\;{\rm{weight}}}^{1}$ and ${{\rm{BigBird}}}_{{\rm{w/o}}\;{\rm{weight}}}^{2}$. We adopted the Linear SVC implementation (https://scikit-learn.org/stable/modules/generated/sklearn.svm.LinearSVC.html) with the default hyperparameters settings to train the SVM classifier on our training dataset. We used majority voting from predictions of all five different (4 BigBird + SVM) models in our ensemble learning setting. We break the ties with predictions from the best-performing classifier. The detailed video classification results are shown in Table 1.

**Table 1:** Performance comparison (on test dataset) of the different video classifiers used in creating MedVidCL dataset.

Model	Precision	Recall	F1-score
${{\rm{BigBird}}}_{{\rm{w/o}}\;{\rm{weight}}}^{1}$	93.27	93.16	93.17
${{\rm{BigBird}}}_{{\rm{w/o}}\;{\rm{weight}}}^{2}$	94.65	92.66	93.60
${{\rm{BigBird}}}_{{\rm{w/}}\;{\rm{weight}}}^{2}$	93.53	91.79	92.62
${{\rm{BigBird}}}_{{\rm{w/}}\;{\rm{weight}}}^{2}$	92.61	92.12	92.36
SVM	94.39	91.13	92.57
Ensemble	**95.07**	**93.65**	**94.33**

**Fig. 4:**
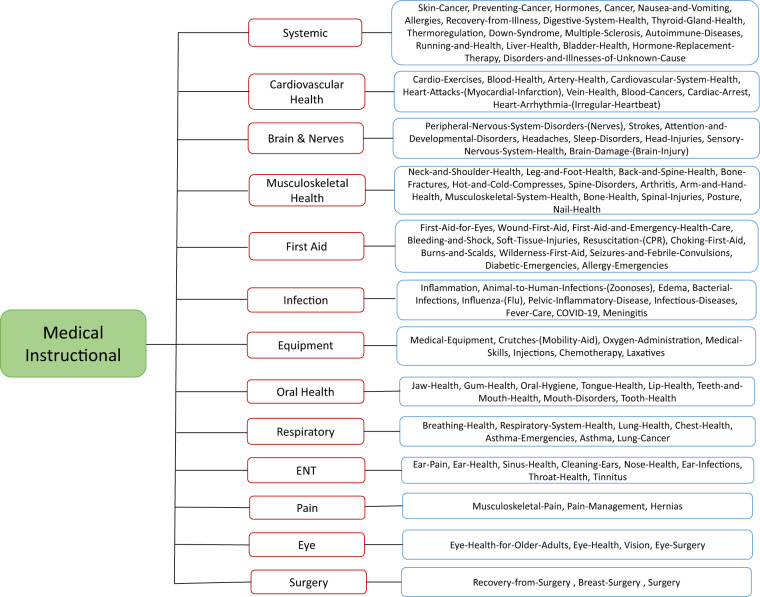
Coarse and fine-grained labels corresponding to the medical instructional category.

All reported results demonstrate the macro average performance.
(C) **Identification of Relevant Videos**: We sampled a subset of videos from the large-scale HowTo100M and YouTube8M^19^ datasets, and we only chose medical and health-related videos from a set of predefined categories marked as appropriate by medical informatics experts. This process yields a collection of 66,312 videos from HowTo100M and YouTube8M datasets.
(D) **Predicting Relevant Video Categories from the Video Classifier**: We utilized the ensemble classifier to predict the category of the relevant videos. The ensemble classifier predicted 13,659 medical instructional videos, 5,611 medical non-instructional videos, and 47,042 non-medical videos from the collection of 66,312 relevant videos.
(E) **Sampling High-Quality Videos and their Manual Assessment**: In order to create a high-quality dataset, we only chose the videos for which the classifier confidence was high for a specific video category and filtered out the videos for which the ensemble classifier confidence was low. In the first step, we filtered out all the videos from the predicted medical-instructional category for which the classifier confidence was below 0.8. A similar strategy was used for medical non-instructional (confidence score below 0.9) and non-medical (confidence score below 0.99). The second and final step involved the manual assessment of the classifier-predicted videos.
(F) **Refining Medical Instructional Videos into the Fine-grained Category**: To further advance the medical instructional category videos, two annotators annotated each video into the wikiHow medical categories (https://www.wikihow.com/Category:Health). Toward this, we provided hierarchical labels for each medical instructional video, specifically coarse-grained and fine-grained labels. We have demonstrated the label hierarchy in Fig. 4, where the root of the tree is the Medical Instructional label, and the first label of the tree are coarse-grained instructional labels. The leaf of the tree exhibits fine-grained instructional labels. In order to create the coarse-grained labels, we manually cluster the fine-grained labels and assign the cluster an appropriate medical category. The MedVidCL dataset contains 13 coarse-grained labels and 110 fine-grained labels for the medical instructional videos.

## Data Records

We have archived a total of nine data records with Open Science Framework (OSF), available at 10.17605/OSF.IO/PC594^13^. The OSF link contains two directories, one for the MedVidCL dataset and the other for the MedVidQA dataset. The MedVidCL directory contains the training, validation, and test split of the MedVidCL dataset. The README file also contains detailed information about each split, including statistics and sample code to process the data and download the videos. Each dataset split is saved in JSON format, which lists data items. Each item in the JSON file contains the relevant key (metadata name) and value (metadata information) pairs, which are *video_id*, *video_link*, *video_title* and *label*. The detailed statistics of the MedVidCL dataset are shown in Table 2. We also archived a sub-directory labeled Med-Instr-Hierarchical in the MedVidCL directory containing JSON files of the training, validation, and test split of the medical instructional video only. Each JSON file contains additional coarse-grained and fine-grained labels describing each video based on video content. We have also provided a CSV file that contains the mapping of each fine-grained label to its coarse-grained label in the sub-directory Med-Instr-Hierarchical. Similar to MedVidCL, we archived another directory for MedVidQA, which also contains the training, validation, and test split of the MedVidQA dataset along with the README file with details about the dataset. Each dataset split is saved in JSON format with data items containing the key-value pairs. The keys to the data items are *sample_id*, *question*, *answer_start*, *answer_end*, *video_length*, *video_id* and *video_url*. The details of each key are provided in the README file. The statistics of the MedVidQA dataset are given in Table 4.

**Table 2:** Detailed class-wise statistics of the MedVidCL dataset.

Video Category	Train	Validation	Test	Total
Medical Instructional	789	100	600	1,489
Medical Non-instructional	2,394	100	500	2,994
Non Medical	1,034	100	500	1,634
Total	4,217	300	1,600	6,117

## Technical Validation

### MedVidQA analysis and validation

In the first step of the MedVidQA dataset creation, we aim to identify and use only trustworthy videos and YouTube channels. A video is categorized as a reliable video if it belongs to a YouTube channel from any of the following sources: **(a)** accredited medical schools with established reputations, **(b)** health institutes, **(c)** health education, **(d)** hospitals, **(e)** medical professionals or experts discussing a particular health-related topic, **(f)** or medical professional appearances and discussions on news channels. We developed an annotation interface (Fig. 10) to select relevant and reliable videos. We have annotated a total of 6,052 YouTube Videos and categorized 1,016 as medical instructional, 2,685 as medical non-instructional, 2,076 as videos from unreliable video/channel, 140 as non-medical videos, and 132 as videos that can not be included in the dataset for other reason. A total of 4 medical informatics experts annotated these videos. To measure the agreements, we sampled 100 videos from the whole collection and asked all the annotators to categorize them into either medical instructional or medical non-instructional categories. We computed the pair-wise inter-annotator agreement (Fig. 6) amongst them using the Cohen’s kappa coefficient^20^, and we found strong agreements (average pair-wise kappa coefficients of 83.75) amongst them.

**Fig. 5:**
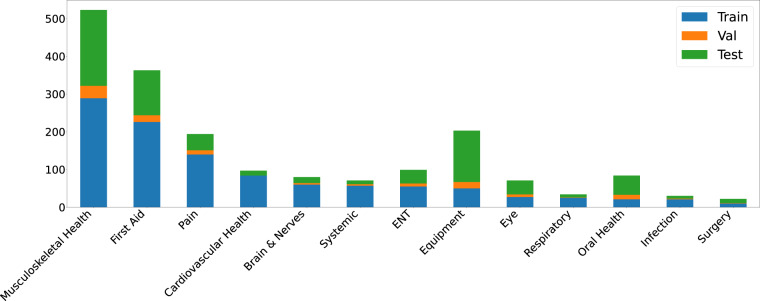
Distribution of the coarse-grained label to the medical instructional category.

**Fig. 6:**
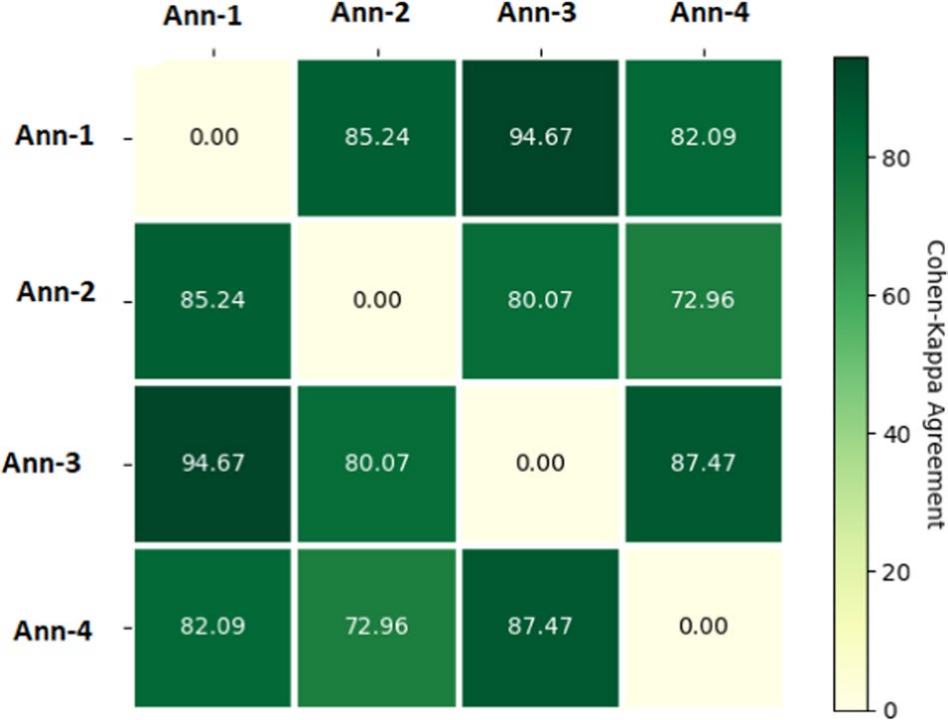
Heatmap for the Cohen’s kappa based inter-annotator agreements.

In the second step of MedVidQA creation, we focus on creating medical or health-related instructional questions. To ease the annotation task, we developed an annotation interface (Fig. 11) to formulate the questions and provide answer timestamps by watching the videos. A total of three medical informatics experts formulated these questions and visual answers. This process yielded a total of 3010 pairs of medical questions and their visual answers from 899 medical instructional videos totaling 95.71 hours. We split the videos into training (800), validation (49), and testing (50) sets. We have provided the detailed statistics in Table 4, Figs. 8, 9. To validate the dataset, we sampled 50 videos and their question-answers annotated by one annotator and asked another annotator to formulate question-answers pairs from the same videos. We first manually assessed whether both the annotators had formulated semantically similar questions from the given video. We then computed the absolute differences between answer timestamps for semantically similar questions formulated by both annotators. With the first assessment, we measured the number of instances where both the annotators had the same agreement on formulating semantically similar questions from the videos. The second assessment validates their agreement on proving the precise and valid answer timestamps from the videos. We found that both the annotators formulated 93 and 96 questions, and 84 out of them were semantically similar. We computed the average absolute difference (AAD) of the start and end timestamps of the visual answers. The AAD values for start and end timestamps are 2.53 and 3.37 seconds, respectively. Lower AAD values signify that both annotators consider almost the same answer timestamps whenever they create a semantically similar question. These assessments validate the quality of the MedVidQA dataset.

**Fig. 7:**
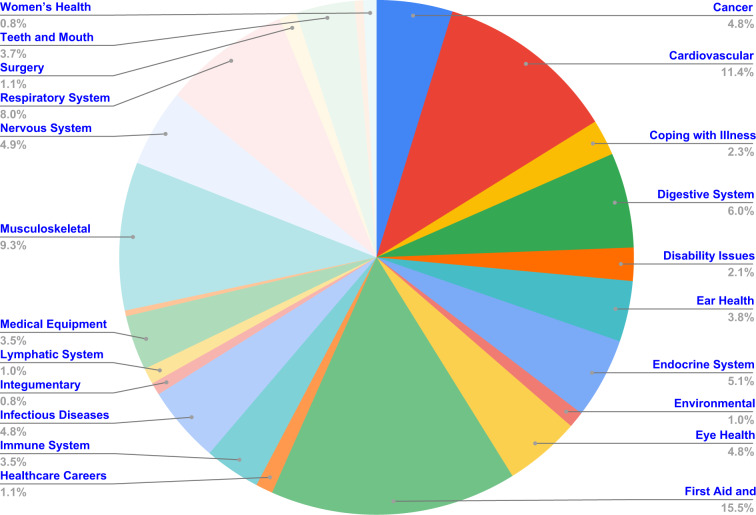
Distribution of the category of the instructional task selected from the wikiHow.

**Fig. 8:**
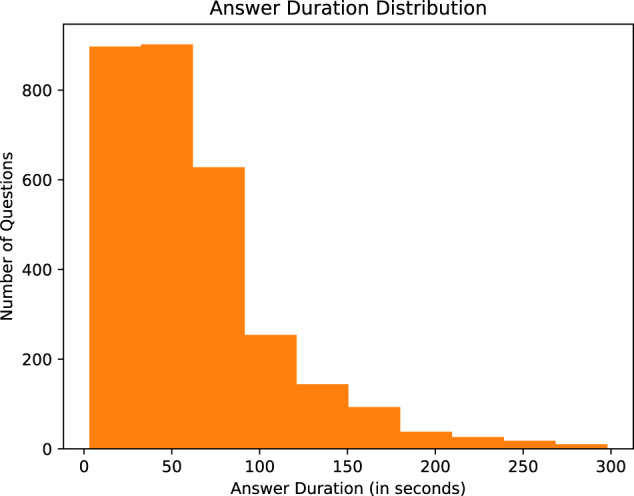
Answer duration distribution in MedVidQA dataset.

**Fig. 9:**
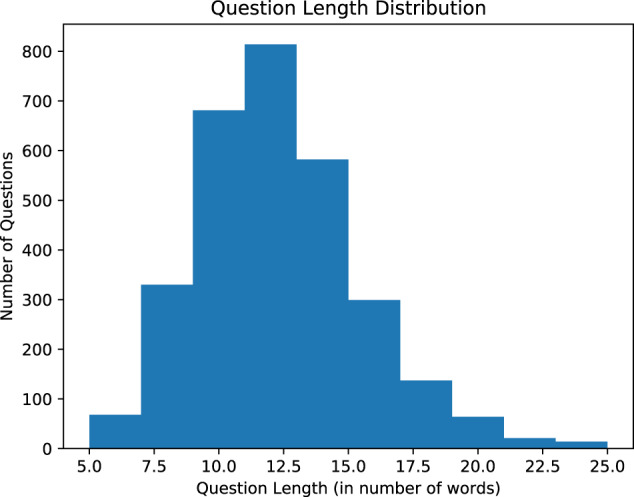
Question length distribution in MedVidQA dataset.

### MedVidCL analysis and validation

To build the MedVidCL dataset, we chose human-annotated (‘*Medical Instructional*’, ‘*Medical Non-instructional*’ and ‘*Non-medical*’) videos from the MedVidQA dataset. We considered this set as the training set for the MedVidCL dataset. To build a validation and test set, we sampled high-confidence videos predicted by a video classifier. To further validate the dataset, we asked the medical informatics expert to review the video category predicted by the model. The expert was asked to *correct* the video category if the video classifier mislabels it and mark the videos as *non-relevant* if there is no conclusive evidence in the videos to label them into any of the video classification categories. The final MedVidCL dataset contains 6,117 videos, amongst which 1,489 are medical instructional, 2,994 are medical non-instructional, and 1,634 are non-medical. We further removed those videos which have a duration longer than 20 minutes. We have provided the dataset’s detailed statistics in Table 2. To analyze the coarse-grained label of the medical instructional category, we have provided the detailed question type distribution in Fig. 5. We found that the majority of the medical instructional videos belong to the coarse-grained categories, “*Musculoskeletal Health* (35.12%)”, “*First Aid* (24.37%)”, “*Equipement* (13.63)” and “*Pain* (13.02%)”. Table 3 shows the coarse and fine-grained class statistics for medical instructional videos in MedVidCL dataset.

**Table 3:** Detailed statistics of the coarse and fine-grained labels of the medical instructional videos in MedVidCL dataset.

Dataset Detail	Train	Validation	Test	Total
Coarse-grained Labels (Max)	3	3	3	3
Coarse-grained Labels (Min)	1	1	1	1
Coarse-grained Labels (Mean)	1.34	1.18	1.14	1.25
Fine-grained Labels (Max)	5	3	4	5
Fine-grained Labels (Min)	1	1	1	1
Fine-grained Labels (Mean)	1.45	1.38	1.2	1.36

**Table 4:** Detailed MedVidQA dataset statistics for questions, videos, and visual answers.

Dataset Detail	Train	Validation	Test	Total
Medical instructional videos	800	49	50	899
Video duration (hours)	86.37	4.54	4.79	95.71
Mean video duration (seconds)	388.68	333.89	345.42	383.29
Questions and visual answers	2,710	145	155	3,010
Minimum question length	5	6	5	5
Maximum question length	25	21	24	25
Mean question length	11.67	11.76	12	11.81
Minimum visual answer length (seconds)	3	10	4	3
Maximum visual answer length (seconds)	298	267	243	298
Mean visual answer length (seconds)	62.29	66.81	56.92	62.23
Proportion of visual answer to the video (%)	15.81	21.10	17.67	16.16
Mode visual answer length (seconds)	34	36	25	34

Question length denotes the number of tokens in the questions after performing tokenization with NLTK^36^ tokenizer.

### MedVidQA benchmarking

We benchmarked the MedVidQA dataset by performing a series of experiments using state-of-the-art natural language video localization approaches. We adopt the proposed architecture^21^, which treats localization of the frames in a video as a span prediction task similar to answer span prediction^22,23^ in text-based question answering. For a given input question and untrimmed video, we first extracted frames (16 frames per second) and obtained the RGB visual features $V=\{{v}_{1},{v}_{2},\ldots ,{v}_{n}\}\in {R}^{n\times {d}_{v}}$ using the 3D ConvNet which was pre-trained on the Kinetics dataset^24^. We also extracted the word representation of the question $\{{w}_{1},{w}_{2},\ldots ,{w}_{m}\}\in {R}^{m\times {d}_{w}}$ using Glove embeddings^25^. As done before^21^, we utilized the character embedding $\{{c}_{1},{c}_{2},\ldots ,{c}_{m}\}\in {R}^{m\times {d}_{c}}$ obtained from a convolutional neural network^26^ to enrich the word representation and obtained the final question representation as $Q=\{{w}_{1}\oplus {c}_{1},{w}_{2}\oplus {c}_{2},\ldots ,{w}_{m}\oplus {c}_{m}\}\in {R}^{m\times \left({d}_{w}+{d}_{c}\right)}$. We encoded the question and video features using the feature encoder, which consists of four convolution layers, followed by a multi-head attention layer^27^. We call the video and question representation obtained from the feature encoder as $\widehat{V}\in {R}^{n\times d}$ and $\widehat{Q}\in {R}^{m\times d}$, respectively. We use the attention flow mechanism^23^ to capture the cross-modal interactions between video and question features.

The attention flow mechanism provides the question-aware video feature representation $\widetilde{V}\in {R}^{n\times d}$. The answers are located using the span predictor as discussed before^21^. Particularly, it uses two unidirectional LSTMs - the first to predict the start timestamp and another to predict the end timestamp of the answer. The first LSTM, labeled LSTM_*s*_, takes the *t*^*th*^ feature ${\tilde{V}}_{t}\in {R}^{d}$ from $\tilde{V}$ to compute the hidden state ${h}_{t}^{s}={{\rm{LSTM}}}_{s}\left({\widetilde{V}}_{t},{h}_{t-1}^{s}\right)\in {R}^{d}$. Similarly the second LSTM, labeled LSTM_*e*_, computes the hidden state ${h}_{t}^{e}={{\rm{LSTM}}}_{e}\left({h}_{t}^{s},{h}_{t-1}^{e}\right)\in {R}^{d}$. Thereafter, scores for the answer start position $\left(S{C}_{t}^{s}=U\times \left({h}_{t}^{s}\oplus {\widetilde{V}}_{t}\right)+b\right)$ and end position $\left(S{C}_{t}^{e}=W\times \left({h}_{t}^{e}\oplus {\widetilde{V}}_{t}\right)+c\right)$ are computed. Here *U* ∈ *R*^2*d*^ (*W* ∈ *R*^2*d*^) and *b* ∈ *R*^2*d*^ (*c* ∈ *R*^2*d*^) are the weight matrices and biases, respectively. Finally, the probability distributions of the start and end positions are computed by ${P}_{s}=softmax(S{C}^{s})\in {R}^{n}$ and ${P}_{e}=softmax(S{C}^{e})\in {R}^{n}$.

The network is trained by minimizing the sum of the negative log probabilities of the true start and end answer position by the predicted distributions *P*_*s*_ and *P*_*e*_ averaged over all samples in the batch. The network trained using the span prediction loss is called video span localization (VSL-BASE). We also experiment with the Query-Guided Highlighting (QGH) technique introduced prior^21^ and call this new network VSL-QGH.

With the QGH technique, the target answer span is considered as the foreground and the rest of the video as the background. It extends the span of the foreground to cover its preceding and following video frames. The extension is controlled by the extension ratio *α*, a hyperparameter. An extended answer span aims to cover additional contexts and help the network focus on subtle differences between video frames. We use the 300 dimensional Glove embeddings and 50 dimensional character embeddings to obtain the word representation in both the VSL-BASE and VSL-QGH networks. We also use 1024 dimensional video features throughout the experiments and hidden state dimensions of 128 in both the LSTM and Transformer-based encoder. The VSL-BASE and VSL-QGH networks are trained using AdamW optimizer^28^ for 30 epochs with an initial learning rate of 0.0001. The best-performing models are chosen based on the performance (IoU = 0.7) on the validation dataset.

#### Benchmarking metrics

We have evaluated the results using **(a)** Intersection over Union (IoU) that measures the proportion of overlap between the system predicted answer span and ground truth answer span, and **(b)** mIoU which is the average IoU over all testing samples. Following a prior protocol^29^, we have used “R@n, IoU = *μ*”, which denotes the percentage of questions for which, out of the top-*n* retrieved temporal segments, at least one predicted temporal segment intersects the ground truth temporal segment for longer than *μ*. In our experiment, we only retrieved one temporal segment; therefore, we have *n* = 1. Following previous studies^21,29^, we have reported *μ* ∈{0.3, 0.5, 0.7} to evaluate the performance of the VSL-BASE and VSL-QGH models.

#### Benchmarking results and discussion

We have performed extensive experiments (*c.f*. Table 5) with VSL-BASE and VSL-QGH models to evaluate the MedVidQA dataset. We start with the Random Mode approach, which randomly predicts the answer span based on the mode value of the visual answer lengths observed in the validation dataset. We also guess the answer span randomly and call the approach Random Guess. We have reported the results of random prediction on the MedVidQA test dataset in Table 5.

**Table 5:** performance comparison of the variants of VSL models on MedVidQA dataset.

Models		IoU = 0.3	IoU = 0.5	IoU = 0.7	mIoU
Random Mode		8.38	1.93	1.21	6.89
Random Guess		7.74	3.22	0.64	5.96
VSL-BASE	FPL 400	19.35	6.45	3.22	18.08
	FPL 600	19.35	10.96	4.51	19.20
	FPL 800	21.93	12.25	5.80	20.15
	FPL 1000	21.93	7.74	3.87	18.86
	FPL 1200	22.58	9.67	5.16	19.97
	FPL 1400	25.16	8.38	4.51	19.33
VSL-QGH		25.81	14.20	6.45	20.12

Here **FPL** refers to the frame position length considered during training the respective models.

With the VSL-BASE model, we ran multiple experiments by varying the frame length from 400 to 1400 to assess its effect on the evaluation metrics. We observe that the VSL-BASE model performs better (except IoU = 0.3) with a frame position length of 800. For IoU = 0.3, an FPL (Frame Position Length) value of 1400 seems to outperform other variants of the VSL-BASE model. With an optimal frame length of 800, we perform our next set of experiments with the VSL-QGH model.

The VSL-QGH models depend on the extension ratio *α*, and the network is trained with join span prediction and visual region (foreground or background) prediction losses. We experimented with the VSL-QGH model by varying the *α* from 0.05 to 0.3 and reported the results in Fig. 12. It can be visualized from Fig. 12 that the model outperforms with *α* = 0.25. We reported the result for the VSL-QGH model with its optimal value of *α* in Table 5. The VSL-QGH model obtained the 25.81 IoU = 0.3, 14.20 IoU = 0.5, 6.45 IoU = 0.7, and 20.12 mIoU. The performance of the VSL-QGH model in terms of mIoU (20.12) is slightly lower (↓0.03) than the VSL-BASE with an FPL value of 800. The results show that visual answer localization is a challenging task, where the model should have the capability of inter-modal communication to locate the relevant frames in the videos. With multiple useful applications of medical visual answer localization in healthcare and consumer health education, we believe that the MedVidQA dataset and benchmarked setup can play a valuable role in further research in this area.

**Fig. 10:**
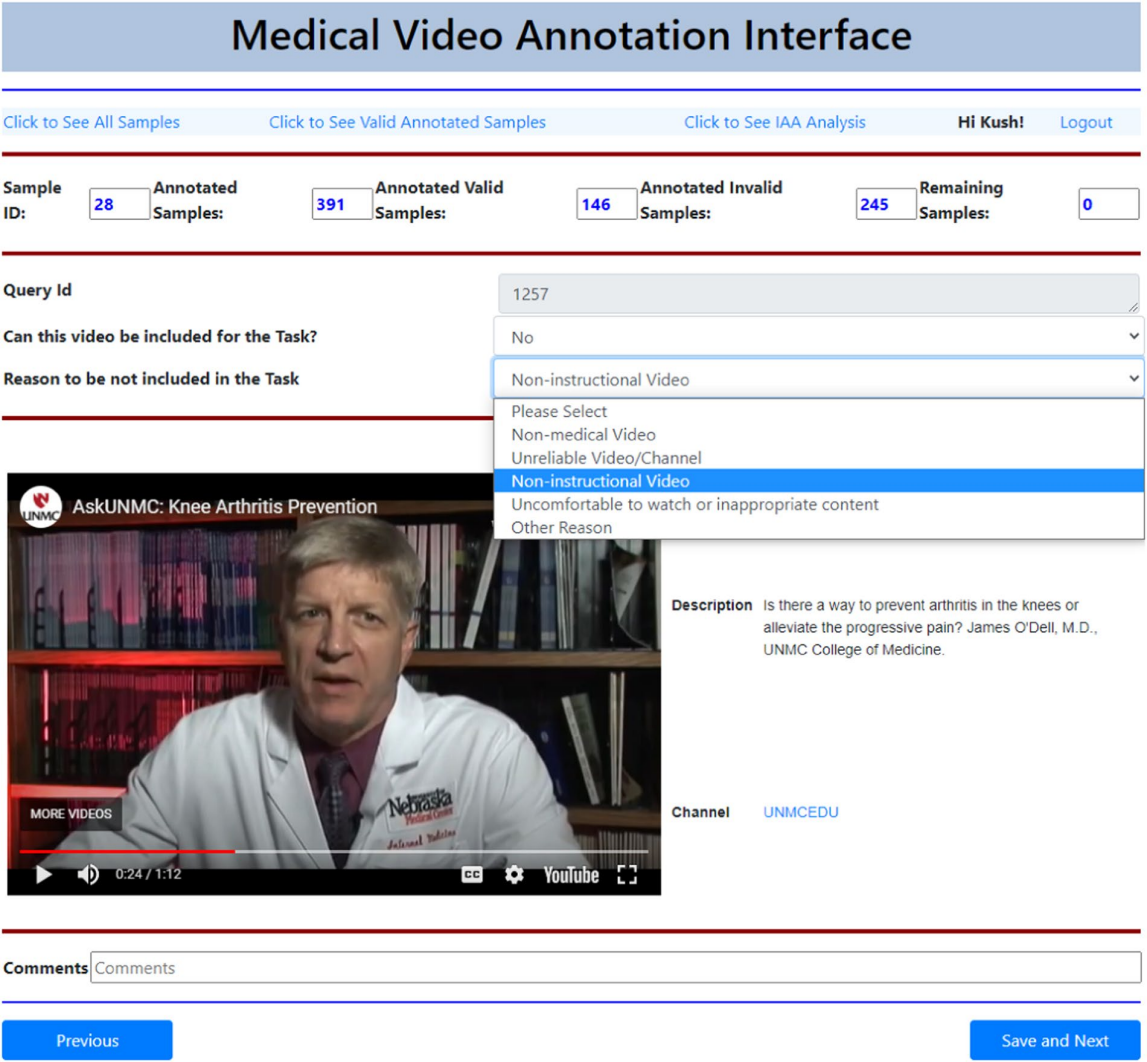
The annotation interface used to label the relevant videos for MedVidQA dataset. It is a hierarchical annotation process, where the annotator needs to provide an appropriate reason if they do not include the given videos for the MedVidQA dataset.

**Fig. 11:**
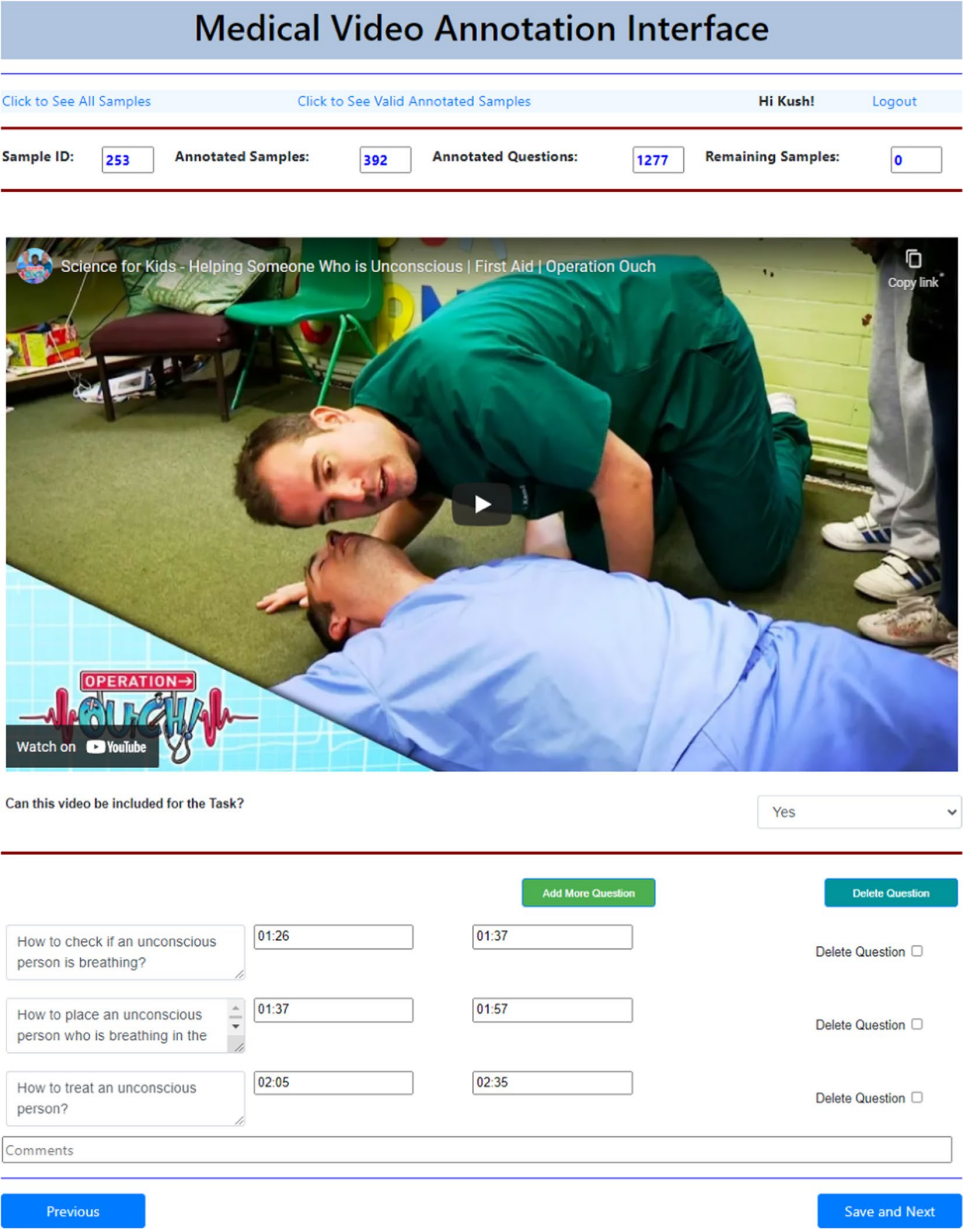
The annotation interface for creating medical and health-related questions and providing answer timestamps in the video.

**Fig. 12:**
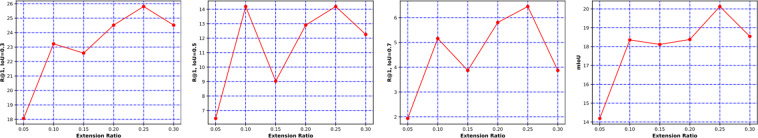
Effect of extension ratio (α) on the performance of VSL-QGH model on MedVidQA test dataset.

### MedVidCL benchmarking

We benchmarked our MedVidCL dataset with multiple monomodal and multimodal approaches. For monomodal approaches, we built several models by considering the language (video subtitle) and vision (video frames) separately for different models. To develop language-based monomodal approaches, we extracted the English subtitles from the videos using the Pytube library (https://pypi.org/project/pytube/). We then trained statistical classifiers such as Linear SVC^30^, and SVM^16^ to predict the video category by considering the language features. Specifically, we first removed common stop words (https://www.nltk.org/book/ch02.html#stopwords_index_term) from the video subtitle to make a clean subtitle text and then train both SVM variants by extracting the TF-IDF features from the clean subtitle text. We have also experimented with pre-trained language models such as BERT-Base-Uncased^31^, RoBERTa-Base^32^, and BigBird-Base^17^. For vision-based monomodal approaches, we extracted 20 frames from each video at a uniform time interval and used these frames as the sequence information to predict the video category. To process these frames, we utilized a 3D ConvNet (I3D), which was pre-trained on the Kinetics dataset^24^, and the resulting features were passed as input to both LSTM and Transformer networks to predict the video category. We have also experimented with the pre-trained (https://huggingface.co/google/vit-base-patch16-224) ViT^33^ model for extracting frames and obtained the frame representation from the ViT feature extractor. Similar to the I3D, we passed the resulting features to LSTM and Transformer networks to predict the video category. We extended our experiments from monomodal to multimodal settings by considering both the language and vision features. Using the language input (video subtitle) and vision input (video frames), we obtained their representations either from LSTM or Transformer networks. We then concatenated the language and vision features together and passed the concatenated features to a feed-forward layer with *softmax* activation to predict the video category. Similar to the monomodal (vision) experiments, we use both I3D and ViT features to perform the multimodal experiments. We also extend the aforementioned experiments to assess the model’s performance on coarse-grained medical instructional videos. Since the prediction of the coarse-grained medical instructional video belongs to the multilabel classification problem, therefore, we replace the *softmax* activation of the classification with *sigmoid* activation function to accommodate all the probable classes given the medical instructional video.

We chose the hyperparameters values based on the classifier performance (average macro F1-score) on the validation dataset. For the Linear SVC and SVM classifiers, the optimal regularization value *C* was 1.5 and 1, respectively. The SVM model with the *sigmoid* kernel outperformed the other kernels on the validation dataset. We utilized the pre-trained Transformer models from Hugging Face^34^ to perform the monomodal (language) experiments. Each pre-trained Transformer model was trained with the AdamW optimizer with the learning rate = 5*e*-5 for 10 epochs and with a batch size of 8 (except for BigBird, where the batch size was 4). The LSTM and Transformer hidden states are set to 128 and 1024, respectively, for all the monomodal (vision) and multimodal experiments. Each monomodal (vision) and the multimodal network was trained with the learning rate = 5*e*-5 for 30 epochs with a batch size of 16. We set the maximum text sequence length for multimodal experiments to 512. For the coarse-grained medical instructional video classification, we trained the mono-models and multimodal and determined the best checkpoint (probability threshold value of 0.22 for *sigmoid* activation) based on the model’s performance (in terms of weighted F1-score) on the validation dataset.

#### Benchmarking metrics

The evaluation metric to assess the performance of the systems are **(a)** F1-score on Medical Instructional Class, and **(b)** macro average F1-score across all the classes. Following the prior work^35^ of multilabel image classification, we evaluated the performance of the system in predicting the coarse-grained medical instructional videos using the metrics mean average precision (mAP) and F1-score.

#### Benchmarking results and discussion

We have provided the detailed results of multiple monomodal and multimodal approaches in Table 6. Among language-based monomodal approaches, BigBird-Base outperforms other methods and achieved 95.68% overall macro average F1-score and 94.28% F1-score for medical instructional class. Pre-trained Transformer-based models perform better than their counterpart SVM variants. Since BigBird can accommodate and handle longer sequences effectively, which is plausible in video subtitle, it shows better performance than the other pre-trained language models (BERT and RoBERTa). Among vision-based monomodal approaches, the feature representation learned using ViT (81.26% overall F1-score) is more effective than I3D (74.43% overall F1-score) with Transformer-based frame sequence processing. With multimodal approaches, we observed improvements over the respective monomodal (vision) approaches. We observed the maximum improvement of 1.12% overall F1-score with the multimodal approach (L + V (ViT) + Transformer) compared to the monomodal (ViT + Transformer) approach. Similar trends are also observed for Medical Instructional video classification, where we reported an increment of 1.12% F1-score with the multimodal approach (L + V (ViT) + Transformer) compared to the monomodal (ViT + Transformer) approach. For the visual answer localization to the health-related questions, it is essential to predict the relevant medical instructional videos correctly; therefore, we prioritize the system’s performance on medical instructional classes compared to the overall video classes. In this case, the F1-score on medical instructional videos is more important than the overall F1-score.

**Table 6:** Performance of the monomodal and multimodal approaches on MedVidCL test dataset.

Models		Precision	Recall	F1-score	Precision (Med-Inst)	Recall (Med-Inst)	F1-score (Med-Inst)
Monomodal (Language)	Linear SVC^30^	89.64	89.71	88.41	99.76	70.33	82.50
	SVM^16^	89.54	88.73	87.42	100.0*	67.00	80.24
	BERT-Base-Uncased^31^	92.82	93.23	92.91	95.98	87.50	91.54
	RoBERTa-Base^32^	94.58	94.98	94.67	97.99	89.33	93.46
	BigBird-Base^17^	95.58*	95.96*	95.68*	98.19	90.67*	94.28*
Monomodal (Vision)	I3D + LSTM^24,37^	75.62	75.88	75.11	81.66	63.83	71.66
	ViT + LSTM^33,37^	82.07^†^	81.16	80.49	89.62^†^	67.67	77.11
	I3D + Transformer^24,27^	75.18	75.41	74.43	83.14	60.83	70.26
	ViT + Transformer^27,33^	81.76	82.06^†^	81.26^†^	89.25	69.17^†^	77.93^†^
Multimodal (Language + Vision)	L + V (I3D) + LSTM	75.96	76.16	75.68	79.68	66.67	72.60
	L + V (ViT) + LSTM	82.57	82.16	81.40	90.22	67.67	77.33
	L + V (I3D) + Transformer	74.74	75.10	74.80	76.23	69.50^‡^	72.71
	L + V (ViT) + Transformer	83.65^‡^	83.12^‡^	82.38^‡^	92.22^‡^	69.17	79.05^‡^

The results shown here are not a comparison amongst the models but show the variety of the models used to benchmark the dataset. Here L and V denotes the Language and Vision, respectively. Precision, Recall, and F1-score denote macro average over all the classes. The best results amongst monomodal (language) approaches are highlighted with the * symbol. Similarly, we show the best monomodal (vision) and multimodal results with the ^†^ and ^‡^ symbols, respectively.

We also experiment with the best language monomodal: **BigBird-Base**, vision monomodal: **ViT + Transformer**, and multimodal: **L + V (ViT) + Transformer** on the test set of the coarse-grained medical instructional videos of the MedVidCL dataset. We reported the results in Table 7 and observed that language monomodal (BigBird-Base) obtained the best performance in terms of mAP and F1 scores. Similar to the behavior of the multimodal approaches analyzed in Table 6, for the coarse-grained labels also, we notice a slight increase in the performance compared to the vision monomodal: **ViT + Transformer**. The detailed class-wise performance of each coarse-grained class is shown in Table 8. The best-performing BigBird-Base model achieves the maximum F-score of 85.11% for the “*Oral Health*” category. “*Musculoskeletal Health*”, “*ENT*”, and “*Brain & Nerves*” are the next few labels where the BigBird-Base model reported considerable performance. With the variety of multiple approaches, our goal is not to establish a state-of-the-art approach for the task rather, we wanted to provide strong baselines and insights on the monomodal and multimodal approaches for the MedVidCL dataset. We believe that a sophisticated language-vision fusion mechanism will further improve the performance (overall F1-score, Medical Instructional F1-score, and Coarse-grained Medical Instructional F1-score) of the multimodal approaches.

**Table 7:** Performance of the best monomodal and multimodal approaches from Table 6 on coarse-grained medical instructional videos of MedVidCL dataset.

Models		mAP	F1 (macro)	F1 (micro)	F1 (weighted)
(1)	BigBird-Base	57.53	47.77	60.32	60.2
(2)	ViT + Transformer	27.2	25.86	43.26	45.53
(3)	L + V (ViT) + Transformer	28.02	26.12	41.39	44.3

Here **(1)**, **(2)** and **(3)** represent the Monomodal (Language), Monomodal (Vision), and Multimodal (Language + vision) models, respectively.

**Table 8:** Class-wise performance (F1-score) on coarse-grained medical instructional videos of MedVidCL dataset using the best monomodel and multimodal models from Table 6.

Models		Pain	Oral Health	Brain & Nerves	Infection	Equipment	ENT	Musculoskeletal Health	First Aid	Eye	Cardiovascular Health	Surgery	Respiratory	Systemic
(1)	BigBird-Base	35.90	85.11	45.16	00.00	44.00	72.97	73.09	64.48	70.18	26.23	00.00	70.59	33.33
(2)	ViT + Transformer	21.79	57.83	4.55	00.00	45.10	22.41	66.25	50.91	14.29	11.63	11.76	26.09	3.51
(3)	L + V (ViT) + Transformer	21.47	55.91	6.45	00.00	45.74	22.07	63.09	50.00	4.35	10.08	21.05	28.57	2.63

## Usage Notes

We have provided detailed instructions in the README file of the Open Science Framework repository (10.17605/OSF.IO/PC594)^13^ describing how to process the MedVidCL and MedVidQA datasets. The source code to process the video and extract the features for building models can be found in the GitHub repository (https://github.com/deepaknlp/MedVidQACL).

### Videos

The videos used to create the MedVidCL and MedVidQA datasets are publicly available on YouTube. We have provided the links to download them along with the other metadata information.

### Video features

For the MedVidQA and MedVidCL datasets, we have extracted the features using a 3D ConvNet-based I3D model that was pre-trained on the Kinetics dataset. Additionally, we have also extracted video features for the MedVidCL dataset using the pre-trained ViT model. We have publicly released these video features, which can be downloaded from our resource page (https://bionlp.nlm.nih.gov/).

## Data Availability

The code to process the MedVidCL and MedVidQA datasets^13^ and reproduce the results of the experimental benchmarks (with hyperparameters values) can be found at https://github.com/deepaknlp/MedVidQACL.
